# Two Subclasses of Differentially Expressed *TPS1* Genes and Biochemically Active TPS1 Proteins May Contribute to Sugar Signalling in Kiwifruit *Actinidia chinensis*

**DOI:** 10.1371/journal.pone.0168075

**Published:** 2016-12-19

**Authors:** Charlotte Voogd, Lara A. Brian, Erika Varkonyi-Gasic

**Affiliations:** The New Zealand Institute for Plant & Food Research Limited (Plant & Food Research) Mt Albert, Auckland Mail Centre, Auckland, New Zealand; University of Nebraska-Lincoln, UNITED STATES

## Abstract

Trehalose metabolism and its intermediate trehalose-6-phosphate (T6P) are implicated in sensing and signalling sucrose availability. Four class I *TREHALOSE-6-PHOSPHATE SYNTHASE* (*TPS1*) genes were identified in kiwifruit, three of which have both the TPS and trehalose-6-phosphate phosphatase (TPP) domain, while the fourth gene gives rise to a truncated transcript. The transcript with highest sequence homology to Arabidopsis *TPS1*, designated *TPS1*.*1a* was the most highly abundant *TPS1* transcript in all examined kiwifruit tissues. An additional exon giving rise to a small N-terminal extension was found for two of the *TPS1* transcripts, designated *TPS1*.*2a* and *TPS1*.*2b*. Homology in sequence and gene structure with *TPS1* genes from Solanaceae suggests they belong to a separate, asterid-specific class I TPS subclade. Expression of full-length and potential splice variants of these two kiwifruit *TPS1*.*2* transcripts was sufficient to substitute for the lack of functional TPS1 in the yeast *tps1Δ tps2Δ* mutant, but only weak complementation was detected in the yeast *tps1Δ* mutant, and no or very weak complementation was obtained with the *TPS1*.*1a* construct. Transgenic Arabidopsis lines expressing kiwifruit *TPS1*.*2* under the control of 35S promoter exhibited growth and morphological defects. We investigated the responses of plants to elevated kiwifruit *TPS1* activity at the transcriptional level, using transient expression of *TPS1*.*2a* in *Nicotiana benthamiana* leaves, followed by RNA-seq. Differentially expressed genes were identified as candidates for future functional analyses.

## Introduction

Trehalose has a central role as an energy source and in stress response in microorganisms and invertebrates [1]. It has an important role as osmotic protectant in bacteria, fungi, archaea and insects [2] and is thought to protect against desiccation in specialized resurrection plant species. However, in most plant species trehalose is accumulated in trace amounts and is therefore unlikely to be important as an osmoprotectant. Instead, trehalose metabolism is implicated in providing a link between plant metabolism and development [3], with its intermediates sensing and signalling carbon availability [4,5]. Alterations in trehalose metabolism cause highly pleiotropic phenotypes, implicating trehalose metabolism in regulation of embryo development, vegetative growth, flowering and architecture [6–12], stomatal conductance [12–14], photosynthesis and starch metabolism [15–17]. Manipulation of trehalose metabolism has also been utilized in model and crop species to increase abiotic stress tolerance [18–21] and changes in transcriptome of transgenic plants expressing *TPS1* were described [22,23].

The biosynthesis of trehalose in plants involves generation of trehalose-6-phosphate (T6P) from UDP-glucose and glucose-6-phosphate (G6P) by trehalose-6-phosphate synthase (TPS), followed by dephosphorylation to trehalose by trehalose-6-phosphate phosphatase (TPP). Trehalose is subsequently hydrolysed by trehalase (TRE) into two glucose residues to actively prevent accumulation of trehalose in higher plants [24]. Mutant and transgenic analyses have demonstrated that changes in T6P concentrations are associated with developmental and metabolic effects observed upon perturbing trehalose metabolism. T6P is therefore proposed to be a signal for sucrose availability and is implicated in sucrose homeostasis in plant cells [25], potentially via the SNF1-related protein kinase (SnRK1) pathway and the transcription factor bZIP11 [3,26–28].

Plant TPS and TPP enzymes are encoded by gene families, while TRE is encoded by a single gene [4]. Each of the Arabidopsis and rice genomes has 11 *TPS* genes, and, respectively, 10 and 13 *TPP* genes, while 12 *TPS* genes and 10 *TPP* genes are reported in the *Populus* and 14 *TPS* and 11 *TPP* in the maize genomes [13,29–31], which have resulted from independent duplication events [2,4]. *TPS* genes are divided into two classes. Class I *TPS* genes are generally present in a single or low number copy, usually encoding catalytically active TPS enzymes with both TPS and TPP domains and inactive phosphatase boxes. Among four Arabidopsis class I *TPS* genes, only *AtTPS1* has previously been shown to encode for TPS activity by expression in the yeast *tps1Δ* mutant [32], but a recent study demonstrated that expression of *AtTPS2* or *AtTPS4* is sufficient for the yeast *tps1Δ tps2Δ* mutant to grow on glucose and accumulate T6P and trehalose [33]. Therefore at least three catalytically active TPS isoforms are found in Arabidopsis. However, only the Arabidopsis *tps1* mutant shows growth defects, suggesting that T6P synthesis may not be the only role of *AtTPS1*. Class II *TPS* genes encode proteins which have both TPS and TPP domains, but lack residues required for interaction with the substrate, do not possess TPS or TPP activity and may instead have a regulatory role [32,34]. All plant *TPP* genes encode proteins with a unique TPP domain and conserved phosphatase domains, and all Arabidopsis TPP enzymes are active in yeast. The similar activity but differential expression patterns suggest specialized function [13].

Most work describing T6P stress response, energy sensing and signalling networks and underlying molecular mechanisms has utilized Arabidopsis, but it is becoming clear that aspects of T6P signalling and role in plant development might be conserved across species [31,35,36], with some caveats. However, there has been little research in trehalose metabolism and signalling in woody perennial plants. Currently, very little is known about the number and structure of *TPS1* genes, or if any of the TPS1 proteins has catalytic activity. This study aimed to identify kiwifruit *Actinidia chinensis* trehalose metabolism genes. Specifically, we aimed to characterize class I *TPS* genes and determine their catalytic activity and expression patterns, followed by evaluation of potential roles in transgenic model plants. Further, we used heterologous transient expression to investigate the response of plants to elevated kiwifruit TPS1 activity.

## Materials and Methods

### Identification of kiwifruit trehalose metabolism genes

Genes were identified using annotation search and reciprocal blast analysis of the kiwifruit genomic sequence database [37] and the Plant & Food Research EST database [38]. Arabidopsis TPS, TPP and trehalose protein sequences were used as tblastn queries. The EST sequences were further used to annotate the TPS genes manually and to devise predicted protein sequences (GenBank accession numbers KX249682-KX249687). Predicted protein sequences were compared with those of Arabidopsis, rice, poplar, tomato and potato using Geneious ClustalW Alignment, and phylogeny was constructed using the Neighbour-joining method in Geneious Tree Builder (Geneious, Biomatters Ltd, version 8.1.2) (http://www.geneious.com) [39].

### Plasmid construction

TPS coding sequences, flanked by Invitrogen™ Gateway™ attL sites, were synthesized by GenScript (Piscataway, NJ, USA) and cloned using Gateway LR Clonase™ (ThermoFisher Scientific, Waltham, MA, USA) into the yeast expression vector pURA3ΔNLS and the plant expression vector pHEX2 [40]. pURA3ΔNLS is based on the pTFT1 [41]-derived Gateway-enabled vector pTFT1GW6, further modified to express the URA3 selectable marker instead of ADE2 (pURA3, constructed by C. Brendolise, Plant & Food Research). The nuclear localisation sequence was spliced out by overlap extension PCR and the resulting fragment cloned into PvuII-digested pURA3. The PCR primer sequences are presented in S1 Table.

### Plant transformation and growth

*Agrobacterium tumefaciens*-mediated Arabidopsis transformation was performed as described [42,43]. *Nicotiana benthamiana* transformation was performed using the leaf disc protocol [44]. Seeds of transgenic plants were selected on half-strength Murashige and Skoog (½ MS) medium supplemented with kanamycin and placed in a growth room under a long day (LD, 21°C, 16/8 h light/dark) regime.

### Yeast complementation assays

The *Saccharomyces cerevisiae* wild-type strain W303-1A (Mata leu2-3, 112 ura3-1 trp1-1 his3-11, ade2-1 can1-100 GAL SUC2) [45] and W303-1A-derived TPS mutant strains were used for all complementation assays. Yeast *TPS1*, *A*. *chinensis TPS1*.*1a*, *AcTPS1*.*2a*, *AcTPS1*.*2a EXT*, *AcTPS1*.*2b* and *AcTPS1*.*2b EXT*, cloned in the pURA3ΔNLS vector (as above), were introduced into the single-deletion strain YSH290 (W303-1A, *tps1*Δ::*TRP1*) [46] and double-deletion strain YSH652 (W303-1A,*tps1*Δ::*TRP1 tps2*Δ::*LEU2*) [47]. A W303-1A wild-type strain carrying the empty pURA3ΔNLS plasmid was used as a positive control, along with *tps1*Δ and *tps1*Δ *tps2*Δ deletion strains complemented with pURA3ΔNLS-*ScTPS1*. Yeast transformation was performed as described [48] and positive transformants were selected on synthetic dropout medium lacking uracil and containing 2% (w/v) galactose. For the complementation assay, starter cultures were grown overnight at 28°C while shaking at 200 rev/min in selective minimal medium containing 2% (w/v) galactose. Six serial dilutions were made, starting from an OD_600_ of 0.5 and 10 μL of each dilution was spotted on plates containing selective medium supplied either with 2% (w/v) galactose or 2% (w/v) glucose. Plates were incubated for 48 h at 30°C and pictures were taken using a digital camera. Proof that any observed complementation was due to the presence of an introduced TPS gene was obtained by confirming loss of complementation after growth under non-selective conditions (synthetic dropout medium including uracil and containing 2% (w/v) galactose) and subsequent selection on FOA-containing medium.

### Tissue sampling, RNA extraction and sequencing

Tissue was sampled from *A*. *chinensis* Planch. ‘Hort16A’ growing at the Plant & Food Research orchard near Kerikeri, New Zealand, in 2015. All sampling was performed from three individual plants (biological replicates), at midday, to avoid variation. Total kiwifruit RNA was isolated using the Spectrum™ Plant Total RNA Kit (Sigma-Aldrich, St. Louis, MO, USA). The genomic DNA was removed and cDNA synthesized using the QuantiTect Reverse Transcription Kit (Qiagen, Hilden, Germany) according to the manufacturer’s instructions. Daily expression analysis was performed on previously described cDNA samples [48].

Leaves of *N*. *benthamiana* plants grown in a containment glasshouse were infiltrated with *Agrobacterium tumefaciens* suspension culture as described [40]. Two young leaves each of three plants were used for each treatment. Six samples were collected by punching ~ 6-mm diameter discs from each treated leaf, and the samples from the same plant were pooled to represent one biological replicate. Infiltration and sample collection were performed at midday, to avoid variation. The RNA was extracted using the Spectrum Plant Total RNA Kit (Sigma-Aldrich) and the quality and quantity of the total RNA were checked using a NanoDrop ND-1000 spectrophotometer (ThermoFisher Scientific) and the Agilent 2100 Bioanalyzer (Agilent Technologies, Santa Clara, CA, USA). Three RNA samples per treatment and time point were used for subsequent library construction and sequencing (three biological replicates per each treatment). The sequencing libraries were constructed according to the TruSeq RNA sample preparation guide (Illumina, San Diego, CA, USA) and subsequently sequenced by an Illumina Genome Analyzer (HiSeq 2000, Illumina) obtaining paired-reads of 100 bp. Library construction and sequencing were performed at Macrogen (Seoul, Republic of Korea).

### Sequence analysis

The resulting RNA-seq reads were passed through the FastQC tool version 0.11.2 (http://www.bioinformatics.babraham.ac.uk/projects/fastqc/) to check the overall quality of the raw data from sequencing. Macrogen TruSeq adapters were clipped from the reads and filtered for a minimum length of 40 bp and minimum quality threshold of 28 using fastq-mcf version 1.04.803 (https://code.google.com/archive/p/ea-utils/). Reads containing unknown nucleotides (Ns) and the first 14 bp of each read were removed using a custom Perl script (written by Cecilia Deng, Plant & Food Research). The remaining high-quality reads were mapped to the genomic sequence of *A*. *tumefaciens* wild-type strain C58 (ftp://ftp.ncbi.nlm.nih.gov/genomes/all/GCA_000092025.1_ASM9202v1) with Bowtie2 version 2.2.5 [49] on default settings to remove reads derived from *Agrobacterium* contamination. Remaining reads were mapped to the draft *N*. *benthamiana* transcriptome version 1.0.1 (ftp://ftp.solgenomics.net/genomes/Nicotiana_benthamiana/annotation/Niben101/) using Bowtie2 on default settings with the paired-end reads mapped end-to-end. Htseq-count version 0.018 (http://www-huber.embl.de/users/anders/HTSeq/doc/count.html) was used to count the number of unique reads that mapped to transcripts using ‘gene’ as the feature type. The resulting count tables were used for downstream analysis in R using the package DESeq2 [50]. Transcripts with P values adjusted for false discovery rates (FDRs) of 10% and adjusted P value <0.05 were considered to be statistically differentially expressed. The fold changes in differentially expressed genes were calculated by comparing the values for TPS1 samples to appropriate time points of GFP samples and significant differentially expressed genes were determined by fold changes ≥1.5 and P adjusted-values <0.05.

### Gene ontology (GO) annotation

Gene annotations were obtained by reciprocal best blast hits of translated *N*. *benthamiana* EST sequences to Arabidopsis amino acid sequences.

The best Arabidopsis (TAIR 10) hit was used for GO term classification, which was performed using PANTHER [51] (http://pantherdb.org/) and interrogation of expression, using the Arabidopsis eFP Browser (http://bbc.botany.utoronto.ca/efp/cgi-bin/efpWeb.cgi) [52].

### Quantitative real-time PCR (qRT-PCR) analysis

Gene-specific primers for qRT-PCR of *A*. *chinensis* and *N*. *benthamiana* genes were designed using Primer3 (v 2.3.4) in Geneious v 8.1.2 (http://www.geneious.com) [39]. RT-PCRs were performed using FastStart DNA MasterPLUS SYBR Green I reaction mix on a LightCycler® 1.5 instrument (Roche) or SYBR Green I Master reaction mix using the LightCycler® 480 System (Roche). Non-template controls were included in each run. Amplification was carried out with an initial denaturing step at 95°C for 5 min, then 40–50 cycles of 95°C for 5 s, 60°C for 5 s, and 72°C for 10 s (1.5 instrument) or 95°C for 5 min, then 50 cycles of 95°C for 10 s, 60°C for 10 s, and 72°C for 20 s (480 instrument). The PCR efficiency for each individual sample was calculated using the LinRegPCR v 2015.3 software [53–55]. The mean efficiency per amplicon was then included in the calculation of relative expression ratios according to the comparative cycle threshold method [56]. Ct values were determined using the second derivative maximum method in the LightCycler® 480 software 1.5.0. For kiwifruit samples, expression of the commonly used reference genes ACT, EF1α, UBC9, and PP2A [38] was analysed using GeNORM software [57] to identify the most stably expressed gene. Similarly, for the *N*. *benthamiana* samples, the PP2A, F-box and L23 genes were included as reference genes [58]. All primer sequences are listed in S1 Table.

## Results

### Identification of *Actinidia TPS1* genes

Name and sequence searches of the kiwifruit draft genome [37] identified 22 *TPS*, 10 *TPP* and a single *TRE* gene (S2 Table). The majority of predicted coding sequences appeared misannotated, often missing large parts of protein coding sequence. A sequence search of the EST database [38] identified candidates for expressed kiwifruit *TPS* genes (S3 Table). At least 27 ESTs representing partial sequences of TPS genes were identified, with representatives of the class I and class II TPS genes. For some genes, multiple near-identical sequences were found, probably reflecting alleles, sequences from different genomes within polyploid genomes, or orthologs from different kiwifruit species. Most kiwifruit TPS predicted proteins included a TPS and a TPP domain, although truncated genes and ESTs were also identified. These are unlikely to be functional and were not investigated further.

**Fig 1:**
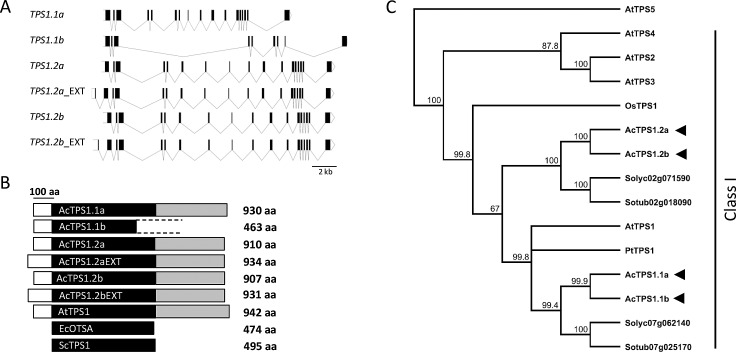
*Actinidia chinensis TPS1* genes. A. *A*. *chinensis TPS1* gene structure. B. *A*. *chinensis* (Ac) TPS1 predicted protein structure compared with TREHALOSE-6-PHOSPHATE SYNTHASE proteins from *Arabidopsis thaliana* (At), *Escherichia coli* (Ec) and *Saccharomyces cerevisiae* (Sc). The TPS and TPP domains are highlighted black and grey, respectively. C. Cladogram of Class I TPS proteins. *A*. *chinensis* (Ac) TPS1 predicted protein sequences (indicated by arrowheads) were aligned with Class I TPS proteins from *Arabidopsis thaliana* (At), *Oryza sativa* (Os), *Populus trichocarpa* (Pt), *Solanum lycopersicum* (Solyc), and *Solanum tuberosum* (Sotub) and a tree was constructed using the Neighbour-joining method and 1000 bootstrap replicates, with Class II AtTPS5 as an outgroup.

Identified putative class I *TPS1* ESTs were used for manual annotation of kiwifruit genome scaffolds and their expression and full-length sequence were further confirmed by subsequent PCR amplification and sequencing. Four kiwifruit *TPS1*-like genes were identified. *TPS1*.*1a*, *TPS1*.*2a* and *TPS1*.*2b* all had 17 exons within the predicted protein coding sequence (Fig 1A) and the first 10 exons encoded the TPS domain, while the remaining seven encoded the TPP domain. *TPS1*.*1b* gene gave rise to a truncated transcript, which lacked part of the TPS and the entire TPP domain, and the sequence of the last exon (as identified by corresponding EST) showed high homology to a downstream gene encoding an ADP-ribosylation factor. Potential alternative splicing resulting in an N-terminal extension and additional short exon was found for both the *TPS1*.*2* transcripts, denoted *TPS1*.*2EXT* (Fig 1A). The alternative translation initiation site context of extended *TPS1*.*2* transcripts appeared favourable [59] and interrogation of the tomato genome (ITAG2.4 gene models at https://solgenomics.net/) revealed that the most similar tomato gene, Solyc02g071590 also contained an additional short exon. The presence of this exon increased the length of the N-terminal extension but did not affect the length of the TPS domain (Fig 1B). Phylogenetic analysis confirmed that all identified kiwifruit predicted TPS1 proteins belonged to the class I TPS subfamily. TPS1.1 sequences share homology with TPS1 proteins from other dicots including Arabidopsis and poplar, while TPS1.2 sequences form a distinct subclade with one of tomato and one of potato TPS1 proteins (Fig 1C).

### Expression analyses

**Fig 2:**
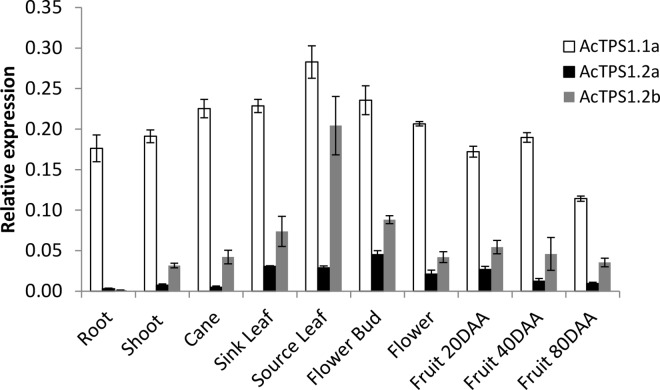
Relative expression of *Actinidia chinensis TPS1* in kiwifruit tissues, normalized to kiwifruit *PP2A*. Error bars represent standard errors (SE) for three biological replicates. DAA, days after anthesis.

Analysis of *Actinidia* ESTs [38] identified *TPS1* expression in young leaf, actively growing shoot buds and developing fruit (S3 Table), consistent with expression in actively growing tissues with high energy demand [32]. qRT-PCR analysis confirmed expression for *TPS1*.*1a*, *TPS1*.*2a* and *TPS1*.*2b* throughout the plant, in all root and shoot tissues analysed (Fig 2). Differential expression levels were detected for three transcripts, with *TPS1*.*1a* showing highest expression. Between *TPS1*.*2* transcripts, *TPS1*.*2b* appeared somewhat more abundant. The same differential expression pattern was also observed in the publicly available mature leaf transcriptome (Sequence Read Archive (SRA) database, accession SRX219918, at http://www.ncbi.nih.gov/sra), and in leaf samples collected over a 24-hour period (S1 Fig), suggesting that *TPS1*.*1a* is the predominant *TPS1* transcript in kiwifruit tissues.

### Functional analyses in yeast

**Fig 3:**
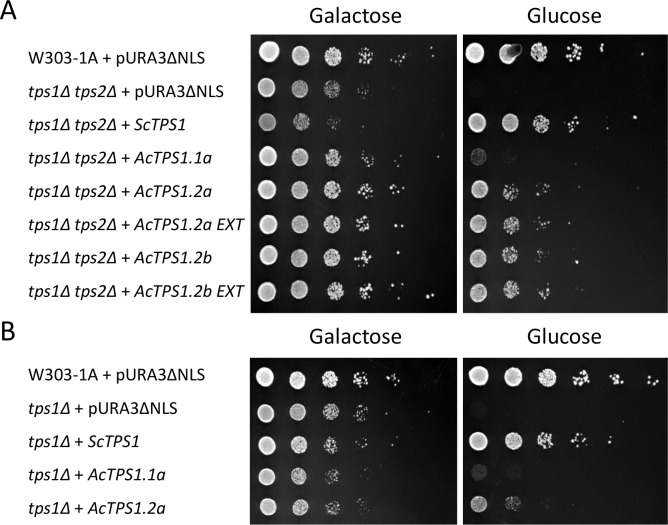
Yeast mutant complementation assays. A. Complementation assay of the yeast *tps1Δ tps2Δ* mutant. B. Complementation assay of the single *tps1Δ* mutant. W303-1A is the wild-type control which can grow on bot galactose and glucose. The mutation results in impaired growth on glucose. Columns represent ten-fold serial dilutions.

Alignment of kiwifruit TPS1 sequences with catalytically active Arabidopsis TPS1, TPS2 and TPS4 identified conservation in amino acid positions important for substrate binding and stabilization of the interaction (S2 Fig). Catalytic activity of kiwifruit TPS1 predicted proteins was further tested in yeast mutants. The disruption of the *TPS1* gene in yeast *S*. *cerevisiae tps1Δ* and *tps1Δ tps2Δ* mutants prevents growth in glucose, but not galactose. The complementation assays in the *tps1Δ tps2Δ* double mutant are considered very sensitive, based on the assumption that the absence of TPP enzyme allows accumulation of T6P even when the introduced TPS1 proteins have only weak activity [33]. The full-length coding sequences of *TPS1*.*1a*, *TPS1*.*2a*, *TPS1*.*2b* and *S*. *cerevisiae TPS1* (*ScTPS1*) were therefore constitutively expressed in the *tps1Δ tps2Δ* yeast strain and assessed for growth on galactose and glucose. *TPS1*.*1b* was not included in the experiment because its coding region was disrupted and it lacked a part of the TPS domain, suggesting it was not functional. *TPS1*.*2* sequences with potential N-terminal extension were also included in the analysis. Expression of all kiwifruit *TPS1*.*2* sequences including the extended transcripts complemented the growth defect on glucose of the yeast *tps1Δ tps2Δ* mutants, confirming catalytic activity and no obvious interference of the N-terminal extension, although serial dilutions of overnight cultures of the different strains suggested that *AcTPS1*.*2* constructs were less effective than *ScTPS1* (Fig 3A). The kiwifruit *TPS1*.*1a* construct resulted in very little growth on glucose (Fig 3A). *AcTPS1*.*1a* and *AcTPS1*.*2a* were also studied for function in the less sensitive yeast *tps1Δ* mutant [32], which lacks the functional TPS enzyme but has TPP activity. *AcTPS1*.*2a* was capable of complementing this mutant, but less effectively than *ScTPS1* (Fig 3B), further confirming catalytic activity, albeit weaker than that of the yeast enzyme. The deliberate loss of the *TPS1*-expressing plasmids in the complementing strains resulted in loss of ability to grow on glucose-containing medium (data not shown), confirming that the complementation occurred as a result of *TPS1* expression and enzymatic activity.

### Phenotypic analyses in model plants

**Fig 4:**
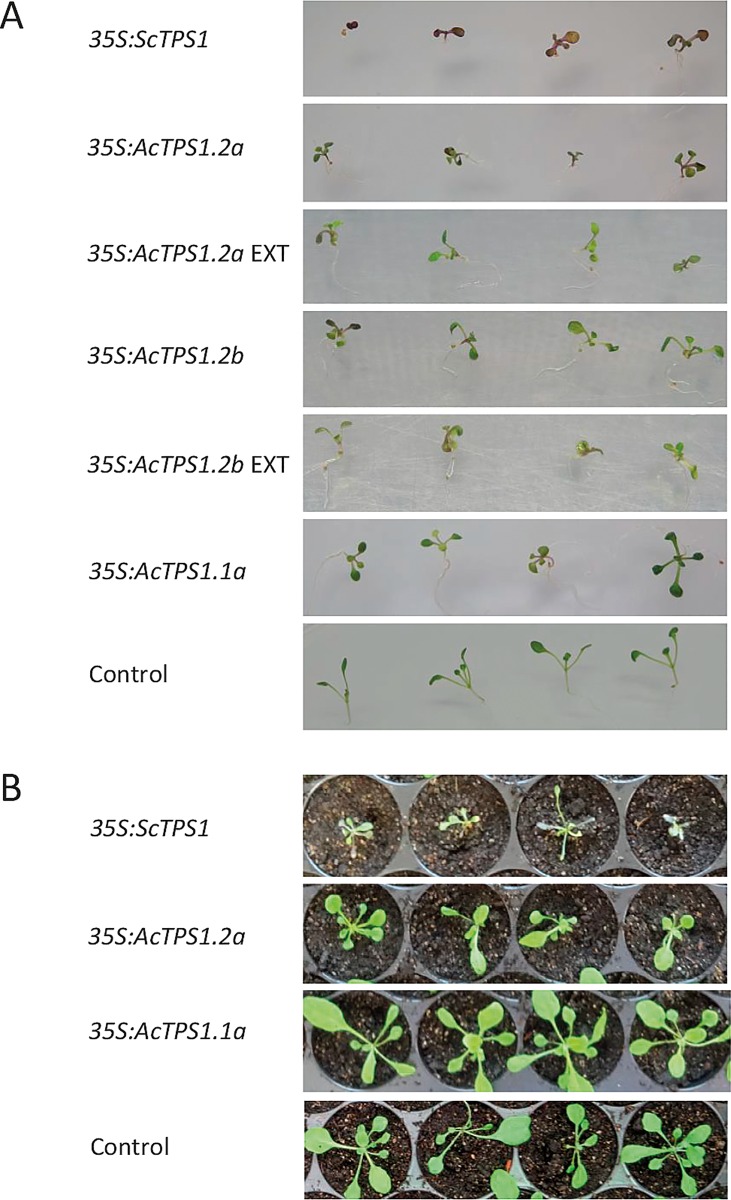
Phenotypes of transgenic Arabidopsis constitutively expressing *TPS1* genes. A. Early growth on ½ MS medium supplemented with sucrose and kanamycin. B. Plants grown in soil.

**Fig 5:**
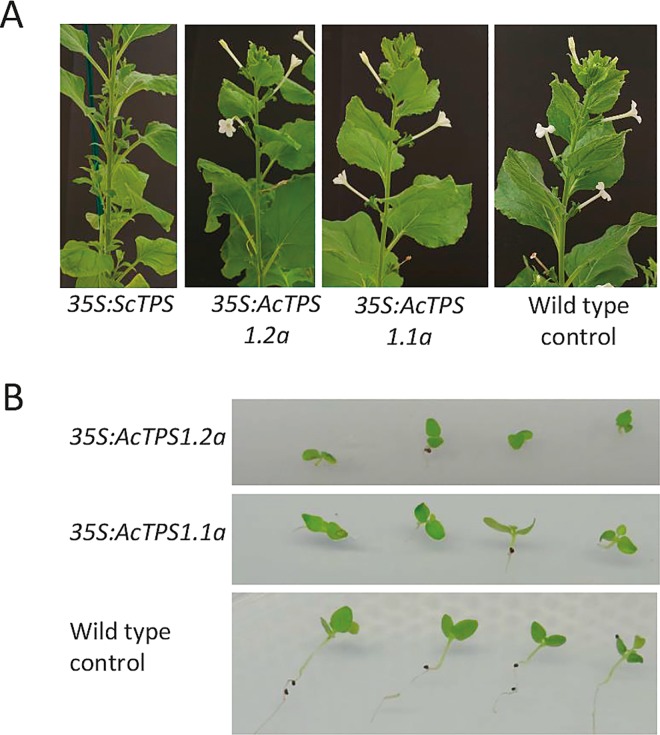
Phenotypes of transgenic *Nicotiana benthamiana* constitutively expressing *TPS1* genes. A. Branching and flowering of representative plants. B. Early growth on ½ MS medium supplemented with sucrose and kanamycin.

Perturbation of trehalose metabolism is associated with developmental changes in plants. Constitutive expression of *ScTPS1* transcript resulted in severely retarded growth and elevated anthocyanin accumulation in Arabidopsis seedlings germinated in tissue culture (Fig 4A). Growth and developmental defects were observed even after transfer to the soil mixture (Fig 4B). Similar but less severe defects were observed in multiple transgenic lines upon constitutive expression of kiwifruit *TPS1*.*2* constructs, although most plants recovered in soil, while constitutive expression of *TPS1*.*1a* gave rise to mostly normal plants (Fig 4). In transgenic *N*. *benthamiana*, constitutive expression of *ScTPS1* transcript resulted in dwarfed plants with extensive branching (Fig 5A); however, kiwifruit *TPS1*.*1a* and *TPS1*.*2a* lines demonstrated slightly slower development, but mostly normal general growth and flowering (Fig 5A and 5B).

### Transcriptome profiling (RNA-seq)

**Fig 6:**
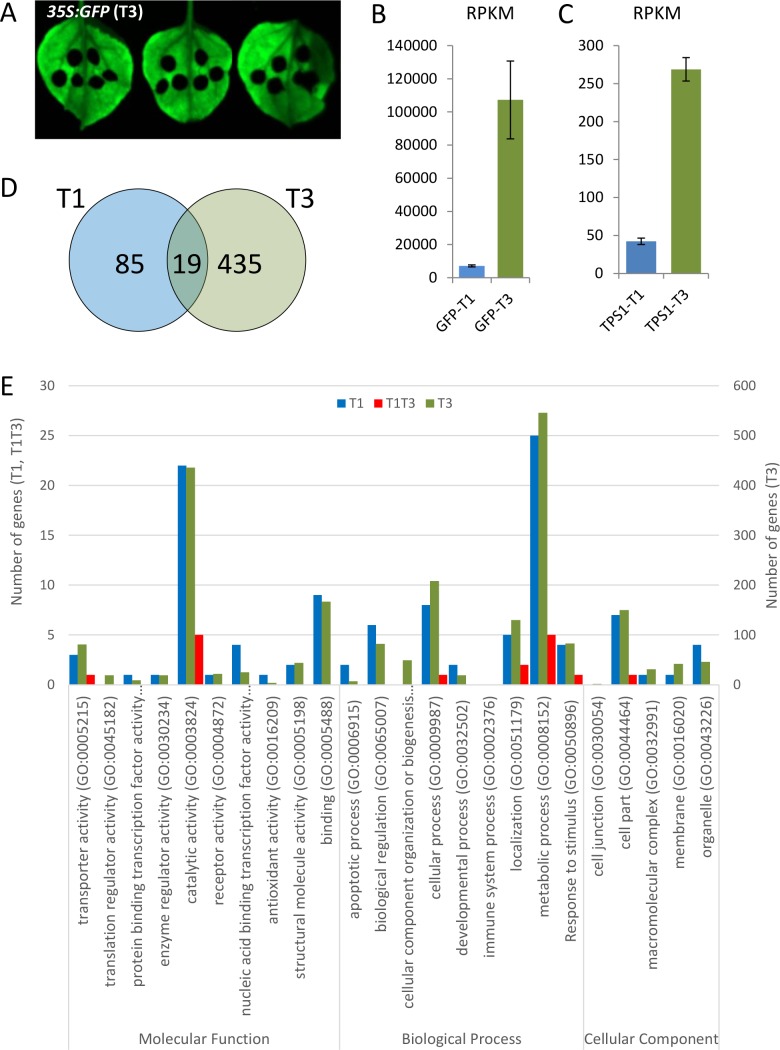
The response of *Nicotiana benthamiana* to transiently elevated kiwifruit *TPS1*.*2a* transcript one day (T1) and three days (T3) after infiltration. A. *Agrobacterium tumefaciens* carrying *GFP* under control of *35S* promoter was used as control. Exposure to UV light confirmed uniform GFP activity, easily detected in all infiltrated leaves after three days. B. Increased *GFP* transcription after infiltration. C. Increased *TPS1* transcription after infiltration. RPKM, frequency counts normalized to number of reads and length of gene. D. Venn diagram showing the number of differentially expressed genes at two sampling times. E. GO term categories of genes with differential expression in response to elevated *TPS1*.*2a*. The graph presents the number of differentially expressed genes between *TPS1*.*2a* and control *GFP* samples identified as significant at T1, T3 and both time points (T1T3).

Whilst *TPS1*.*1a* is the predominant transcript in kiwifruit tissues, the yeast complementation results in the double and single mutants, combined with over-expression phenotypes in Arabidopsis, prompted us to focus on the *TPS1*.*2a* gene as a representative kiwifruit *TPS1*. We also chose this gene as a representative of a potentially asterid-specific clade of *TPS1* genes. To study the immediate plant responses to elevated *TPS1*.*2a* activity, transient expression in *N*. *benthamiana* leaves followed by transcriptome analyses were used to identify differences in gene expression. *Nicotiana* was chosen as a model plant, because (i) agroinfiltration leaf assay is performed easily and efficiently, and (ii) the *TPS1*.*2a* over-expression phenotype is not severe, and therefore the transcriptome changes are less likely to be driven by dramatic changes in sucrose concentrations or by a highly elevated stress response. In addition, *Nicotiana* is a representative of the asterid clade of core eudicots, to which kiwifruit also belongs. Kiwifruit *TPS1*.*2a* under the control of *35S* promoter was delivered by infiltration of *Agrobacterium* suspension culture. *Agrobacterium* carrying *GFP* under control of *35S* promoter was used as a control. RNA was extracted from leaf discs surrounding the infiltration area (Fig 6A) one (T1) and three days (T3) after infiltration and subjected to RNA-sequencing analysis on the Illumina platform.

A total of 12 libraries were prepared to include three biological replicates for each treatment and time point. A total of 396,418,395 pair-end raw reads were generated, ranging from 28,277,533 to 35,938,674 per library (S4 Table). After cleaning and quality checks, reads of each RNA library (ranging from 27,608,067 to 34,933,127 per library; S4 Table) were subjected to a principal component analysis (PCA), which displayed good separation between sample sets (S3 Fig). Only a small number of reads (ranging from 0.06 to 0.19% per library; S4 Table) mapped onto *Agrobacterium* C58 sequence and these reads were removed before mapping of the remaining reads to the *N*. *benthamiana* draft transcriptome at average alignment rate of 84.38% (ranging from 76.64 to 87.36%; S4 Table). Frequency counts were generated for *GFP* and *TPS1*.*2a* sequences and normalized to the number of reads and the length of the gene (RPKM) to demonstrate that both sequences can be detected as perfect matches, and their abundance increased between the sampling points (Fig 6B and 6C).

After one day, 104 genes were found to be significantly differentially expressed between *TPS1*.*2a* and control *GFP* samples (S5 Table). This number increased to 454 three days after infiltration (S6 Table). Among identified transcripts, 74 and 30 were upregulated and 347 and 107 downregulated after one and three days, respectively, with only 19 differentially expressed genes in common for the two time-points (Fig 6D). These results were validated by qRT-PCR analysis of a subset of differentially expressed genes. High correlation between RNA-seq and qRT-PCR results confirmed the accuracy and reproducibility of the transcriptome analysis (S4 Fig). Classification of the differentially expressed sequences identified genes that have been associated with a range of functions, with catalytic and transporter activity identified in the common gene set (Fig 6E). Blast analysis of the common set identified Arabidopsis homologs, which included transcription factors and genes with roles in stress response and hormonal signalling (Table 1). Genes in this set often belonged to larger gene families with multiple members responding to TPS1 activity either at T1, T3 or both.

**Table 1:** Differentially expressed genes between *TPS1*.*2a* and control *GFP* samples identified as significant at both time points (T1 and T3).

*Nicotiana benthamiana* transcript ID	Arabidopsis top hit	e-value	Description (TAIR)	Predicted function (TAIR)	Induced by treatment (eFP browser)	log2Fold (T1)	log2Fold (T3)
Niben101Scf02537g06002.1	AT4G08950	1.00E-158	EXO, EXORDIUM	Response to brassinosteroid	Wounding, oxidative stress, brassinosteroids	1.33	1.05
Niben101Scf01911g03001.1	AT5G60680	5.00E-58	UNKNOWN	Associated with carbohydrate metabolism	Cold stress, ABA	1.3	0.6
Niben101Scf00995g00005.1	AT5G60680	1.00E-54	UNKNOWN	Associated with carbohydrate metabolism	Cold stress, ABA	1.21	0.62
Niben101Scf07244g00001.1	AT4G14130	1.00E-128	XYLOGLUCAN ENDOTRANSGLUCOSYLASE	Cell wall biogenesis and organization	GA inhibitors, brassinosteroid inhibitors	1.01	1.02
Niben101Scf02537g05005.1	AT4G08950	1.00E-158	EXO, EXORDIUM	Response to brassinosteroid	Wounding, oxidative stress, brassinosteroids	0.99	0.89
Niben101Scf03240g01008.1	AT1G66180	0.00E+00	PUTATIVE ASPARTIC PROTEASE	Response to light and ascorbate	Brassinosteroids	0.98	0.72
Niben101Scf02303g00022.1	AT2G39380	0.00E+00	ATEXO70H2, EXO70H2	Vesicle docking involved in exocytosis	ACC treatment	0.93	0.75
Niben101Scf04082g02014.1	AT1G26800	6.00E-50	RING/U-BOX SUPERFAMILY PROTEIN	Zinc ion binding	Heat	0.8	-0.62
Niben101Scf00872g03005.1	AT1G13260	1.00E-129	ETHYLENE RESPONSE FACTOR 4, RAV1	Negative growth regulator	Cold stress	0.8	0.71
Niben101Scf00963g04011.1	AT1G54740	1.00E-24	UNKNOWN	Associated with carbohydrate metabolism	Genotoxic stress	0.79	0.73
Niben101Scf01002g01001.1	AT3G58120	1.00E-60	BZIP61	Transcription factor	Genotoxic and oxidative stress, brassinosteroids, IAA	0.78	1.01
Niben101Scf03506g03001.1	AT3G07650	1.00E-130	BBX7, COL9, CONSTANS-LIKE 9	Regulation of flowering time	Cold stress	0.78	1.16
Niben101Scf14755g00001.1	AT3G01640	1.00E-127	GLUCURONOKINASE, ATGLCAK, GLCAK	Cell wall biogenesis	N/A	0.78	0.74
Niben101Scf02783g01002.1	AT2G36050	7.00E-41	OVATE FAMILY PROTEIN 15, ATOFP15	Negative regulation of transcription	GA3, genotoxic stress	0.74	0.62
Niben101Scf02406g04044.1	AT2G15890	3.00E-66	MATERNAL EFFECT EMBRYO ARREST 14	Pollen tube guidance	N/A	0.68	0.9
Niben101Scf08341g10005.1	AT3G24520	2.00E-98	HEAT SHOCK TRANSCRIPTION FACTOR C1	Regulation of transcription	Cold stress, ABA	0.62	0.76
Niben101Scf01198g02006.1	AT3G02910	7.00E-54	AIG2-LIKE (AVIRULENCE INDUCED GENE)	Involved in response to karrikin	Osmotic and salt stress	0.59	0.79
Niben101Scf12318g00007.1	AT3G54950	1.00E-114	PATATIN-LIKE PROTEIN 6	Acyl-CoA hydrolase activity	Cold and drought stress	-0.67	0.65
Niben101Scf01388g00004.1	AT4G02390	0.00E+00	POLY(ADP-RIBOSE) POLYMERASE 2	Post-translational modification	Genotoxic stress	-0.7	-0.73

## Discussion

### Conservation and divergence of the Class I TPS pathway in kiwifruit

The kiwifruit genome encodes for multiple TPS and TPP proteins and possibly only a single TRE enzyme, similar to previous reports for Arabidopsis, rice, maize and poplar. Multiple class I *TPS* genes have been identified, including a probable non-functional, truncated *TPS1*.*1b*, which is, however, expressed, and a regulatory role cannot be excluded. The *TPS1*.*1a* gene is most similar to Arabidopsis *TPS1* and has a conserved gene structure with 17 exons within the protein coding region. The structure of *TPS1*.*2* genes is potentially different. At this stage, it is not fully clear if the additional intron is placed in the untranslated 5´ UTR, thus resembling the structure of Arabidopsis *AtTPS1*, which also has a very large additional intron in the 5´ UTR (but not *AtTPS2-4*), or if it gives rise to a different variant with an additional exon, not yet reported in other plant species. Very little is known about *TPS1* genes in the asterids, but interrogation of the tomato genome (https://solgenomics.net/) revealed that two of tomato *TPS1* genes also have different structures. Solyc07g062140 coding region contains 17 exons, while a very large and an additional small intron were identified in the 5′ UTR. In contrast, Solyc02g071590 coding sequence contains an additional 5′-terminal short exon, thus resembling kiwifruit *AcTPS1*.*2 EXT* structure. The two *TPS1*.*1* and two *TPS1*.*2* genes are probably the result of a recent genome duplication event. It has been proposed that *A*. *chinensis* underwent two recent whole-genome duplication events, believed to have occurred after the divergence of kiwifruit from tomato and potato [37]. The finding that tomato and potato each have two class I *TPS* genes that cluster separately from each other and share high similarity in sequence and potentially gene structure with kiwifruit *TPS1*.*1* and *TPS1*.*2*, respectively, would suggest that one of these duplications occurred in a much earlier stage of asterid evolution, before the divergence of Solanales, to which tomato and potato belong, from the basal asterid Ericales, to which *Actinidia* belongs.

Neither of the kiwifruit *TPS1* genes appeared to encode truncated forms lacking the auto-inhibitory N-terminal domain and the length of this domain did not seem to affect the functionality. Kiwifruit TPS1 proteins were catalytically active and could complement the yeast *tps1Δ tps2Δ* strain, although TPS1.1a showed extremely weak activity. Complementation of the yeast *tps1Δ* strain was generally more difficult and required multiple screens, which confirmed TPS1.2a activity. Differential complementation was previously reported for class I Arabidopsis *TPS* transcripts, with *TPS1* complementing both mutants, but *TPS2* and *TPS4* complementing only the double mutant, which enables screening for weaker TPS activity for its lack of capacity to convert T6P into trehalose [33]. It has been suggested that Arabidopsis TPS1 has an additional, non-catalytic function which may have an important physiological role, in line with the finding that yeast TPS1 protein rather than its metabolic product provides tolerance to stress and is essential for energy homeostasis and yeast cellular integrity [60]. The lack of natural mutants in kiwifruit and the slow transformation process hinders efforts to establish which of the TPS1 isoforms might have the same dominant physiological role in this species, and future studies in model asterids, particularly tomato, may be better suited to reveal the roles of TPS1 subclades. However, constitutive expression of *TPS1*.*2* resulted in more prominent arrest of growth and increased anthocyanin accumulation in transgenic Arabidopsis, comparable to phenotypes reported for Arabidopsis seedlings expressing *E*. *coli TPS* from the *35S* promoter [11], albeit the effect was less severe than that observed with yeast *ScTPS1*. This result, combined with lower levels of expression in kiwifruit, could mean that TPS1.2 also has higher activity *in planta*. On the other hand, *TPS1*.*1a* is most similar to Arabidopsis *AtTPS1* and is most highly expressed, but appears to have a very weak catalytic activity. Therefore, we cannot exclude the possibility of it having a non-catalytic function in kiwifruit, similar to that proposed for *AtTPS1*.

### Transcriptional changes with elevated TPS1 activity

*TPS1*.*2* was transiently expressed in *N*. *benthamiana* leaves, enabling us to observe the effects of short-term changes in the availability of kiwifruit *TPS1* transcript, and therefore probable changes in TPS1 protein and T6P. Infiltration of *Agrobacterium* suspension expressing GFP showed no obvious adverse effect on leaf physiology: GFP was detected in the whole leaf (outside the point of infiltration) and a steady increase in *GFP* transcript was detected in all biological replicates. Expression of *TPS1* followed the same pattern, but the number of reads was lower and the rate of increase between time points slower, potentially suggesting *TPS1*-related adverse effects.

Most of the identified differentially expressed genes appeared to be involved in a very early (T1) or later response (T3), with a relatively small number (19) demonstrating sustained response to *TPS1*.*2a*. As expected, infiltration of *Agrobacterium* suspension culture itself resulted in large transcriptional changes, demonstrated by the separation of samples between T1 and T3. This large background response may explain the relatively small number of detected differentially expressed genes. Overall, the transcriptomic analyses demonstrated that more genes exhibited significant differences in expression as part of the later response to *TPS1*.*2a*, potentially reflecting downstream effects of metabolic changes resulting from perturbed trehalose signalling. As expected, changes in a range of GO categories were observed with elevated *TPS1*, but the range narrowed in the set of genes consistently up- or downregulated between the two time-points. Analysis of this common set identified that most showed homology to Arabidopsis genes implicated in abiotic stress responses and hormonal signalling.

Several transcription factor gene families were identified with multiple members showing altered expression induced early and/or late by *TPS1*.*2*, and with at least one member in the common set. These included APETALA2/Ethylene Responsive Factor (AP2/ERF) transcription factors, which act as mediators of stress responses and developmental programmes [61] and CO-like transcription factors [62], with roles in growth and developmental processes that include seedling photomorphogenesis, photoperiodic regulation of flowering, shade avoidance, and responses to biotic and abiotic stresses. A transcript with homology to Arabidopsis *bZIP61* and *bZIP34* was also consistently upregulated. While it is proposed that Arabidopsis TPS1 exerts its function via bZIP11, these bZIP transcription factors belong to a different group and are postulated to mediate stress response, e.g. boron deprivation [63,64]. Transcription factors in other classes, e.g. bHLH, DOF, MYB and WRKY, were also differentially expressed in one or both time points, suggesting a major impact of T6P signalling on transcription.

A set of genes induced by *TPS1*.*2* showed homology to *EXORDIUM* (*EXO*), which has a role in meristem function in Arabidopsis [65], cell expansion and brassinosteroid (BR)-mediated responses [66] and growth during low carbon and energy-limiting conditions [67]. More recently, it was demonstrated that apoplastic EXO protein modifies intracellular sucrose and trehalose responses and thus connects the extracellular C status to growth [68]; trehalose feeding induced Arabidopsis *EXO* expression, and therefore a potential increase in trehalose synthesis after *AcTPS1*.*2* infiltration might be the underlying cause for elevated *Nicotiana EXO* expression.

Other differentially expressed gene families identified in this study included genes encoding several classes of F-box proteins, implicated in hormone and stress signalling [69–72] and multiple small auxin-up RNA (SAUR) genes. These proteins are localised in the membrane and cytoplasm [73,74], associated with elongating tissues [73–76] and can inhibit synthesis of auxin and proteins for polar auxin transport [77,78]. In addition to auxin, other hormones and environmental signals affect SAUR gene expression, and it has been postulated that SAURs have a key role in integrating hormonal and environmental signals into distinct growth and developmental responses [79]. A possible mechanism involves activation of plasma membrane H+-ATPases [80], to regulate cell wall-modifying enzymes, including expansins, and thus facilitate uptake of solutes to drive cell expansion. Indeed, a number of *Nicotiana EXPANSIN* genes were downregulated in the early response to *AcTPS1*.*2* infiltration. Similarly, differential expression of other auxin-related transcripts was detected as part of the late response.

**Fig 7:**
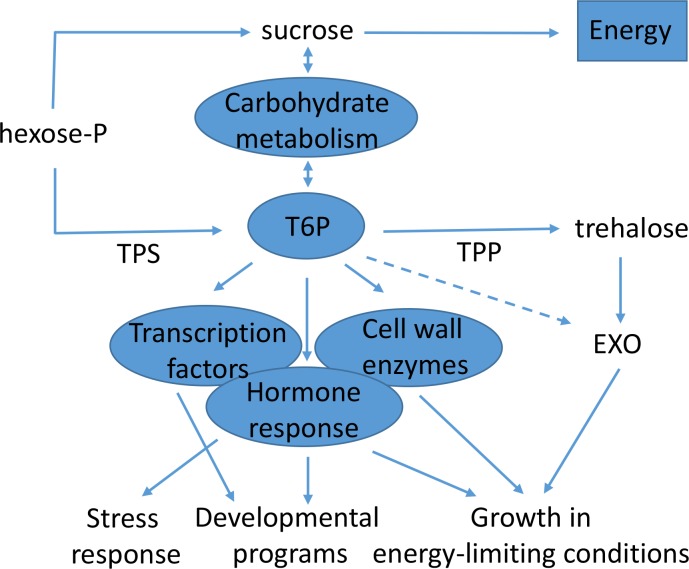
A model for *TPS1* action in *Nicotiana benthamiana* leaf. T6P signalling modulates transcription, hormone signals and cell wall biogenesis to coordinate growth, development and stress responses with carbon availability.

Based on the RNA-seq analysis, we propose a model of *TPS1* action in *N*. *benthamiana* leaf (Fig 7). Given the nature of heterologous transient overexpression, the changes would reflect the *Nicotiana*-specific responses and a comparison with overexpression of *Nicotiana TPS1* would be required to identify potential kiwifruit *TPS1*-specific effects. Similarly, the responses reflect increased accumulation of T6P intermediate and perturbance of trehalose metabolism, but likely include indirect effects resulting from high accumulation of the TPS1 protein. As an example, it is well established that TPS1 activity is regulated post-translationally by phosphorylation at multiple sites [81], hence a high availability of TPS1 protein could occupy kinases to such an extent that they are insufficiently active on other substrates in the plant. Therefore, some of the transcriptome changes detected by RNA-seq may be a consequence of depleted kinase function rather than the TPS1 function.

## Conclusions

Multiple biochemically active TPS1 proteins and differentially expressed *TPS1* genes might contribute to sugar signalling and regulation of kiwifruit growth and development. They fall into two subclasses, one of which is common in eudicots and one of which is found in asterids. Transiently elevated kiwifruit *TPS1*.*2* activity in *Nicotiana* leaf affects transcription of a large number of genes which affect plant growth, development, responses to stress, hormonal signalling and sugar metabolism.

## Supporting Information

S1 Fig Relative expression of *Actinidia chinensis TPS1* in kiwifruit *A*. *chinensis* ‘Hort16A’ source leaves over the 24-hour period. Variation in *Actinidia TPS* transcript accumulation in leaves. Relative expression of kiwifruit *TPS1*.*1*, *TPS1*.*2* and *CONSTANS*-like (*AcCO*, GenBank accession number FG518975) during the day and night cycle, normalized to kiwifruit *PP2A*. Error bars represent SE for three replicate reactions performed on a pooled sample (three plants). Shading represents nigh-time.
(PDF)

S2 Fig Alignment of kiwifruit TPS1 sequences with catalytically active Arabidopsis TPS1, TPS2 and TPS4 identified conservation in amino acid positions considered important for substrate binding and stabilization of the interaction [33]. Residues important for Glc6P and UDP-Glc binding are indicated by green and yellow boxes, respectively, and residues important for stabilization of the interaction are indicated by asterisks.
(PDF)

S3 Fig Principal component analysis (PCA) of RNA-seq libraries. (PDF)

S4 Fig Validation of RNA-seq analysis by qRT-PCR. A random selection of differentially expressed transcripts were amplified using sequence-specific oligonucleotide primers. A. Transcripts identified as upregulated in response to elevated *TPS1*.*2a*. B. Transcripts identified as repressed in response to elevated *TPS1*.*2a*.
(PDF)

S1 Table Oligonucleotide primers used in this study. (XLSX)

S2 Table *Actinidia chinensis* gene models with homology to genes involved in trehalose metabolism. (XLSX)

S3 Table *Actinidia* ESTs with homology to *TPS* genes. (XLSX)

S4 Table RNA-seq workflow. (XLSX)

S5 Table Statistically significant differentially expressed genes one day post infiltration. (XLSX)

S6 Table Statistically significant differentially expressed genes three days post infiltration. (XLSX)
