# Supplementary figures and images for: Two Subclasses of Differentially Expressed TPS1 Genes and Biochemically Active TPS1 Proteins May Contribute to Sugar Signalling in Kiwifruit Actinidia chinensis
Source: PLoS One. 2016 Dec 19;11(12):e0168075. doi: 10.1371/journal.pone.0168075 (PMC5167275; doi:10.1371/journal.pone.0168075)

S1 Fig

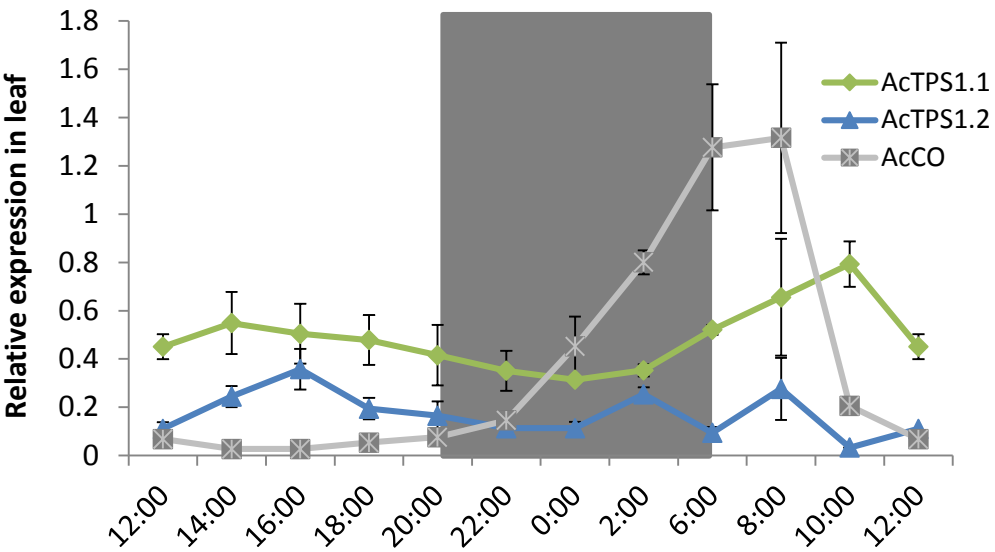

Supplement: S1 Fig — Variation in Actinidia TPS transcript accumulation in leaves. Relative expression of kiwifruit TPS1.1, TPS1.2 and CONSTANS-like (AcCO, GenBank accession number FG518975) during the day and night cycle, normalized to kiwifruit PP2A. Error bars represent SE for three replicate reactions performed on a pooled sample (three plants). Shading represents nigh-time. (PDF) [file pone.0168075.s001.pdf]

S3 Fig

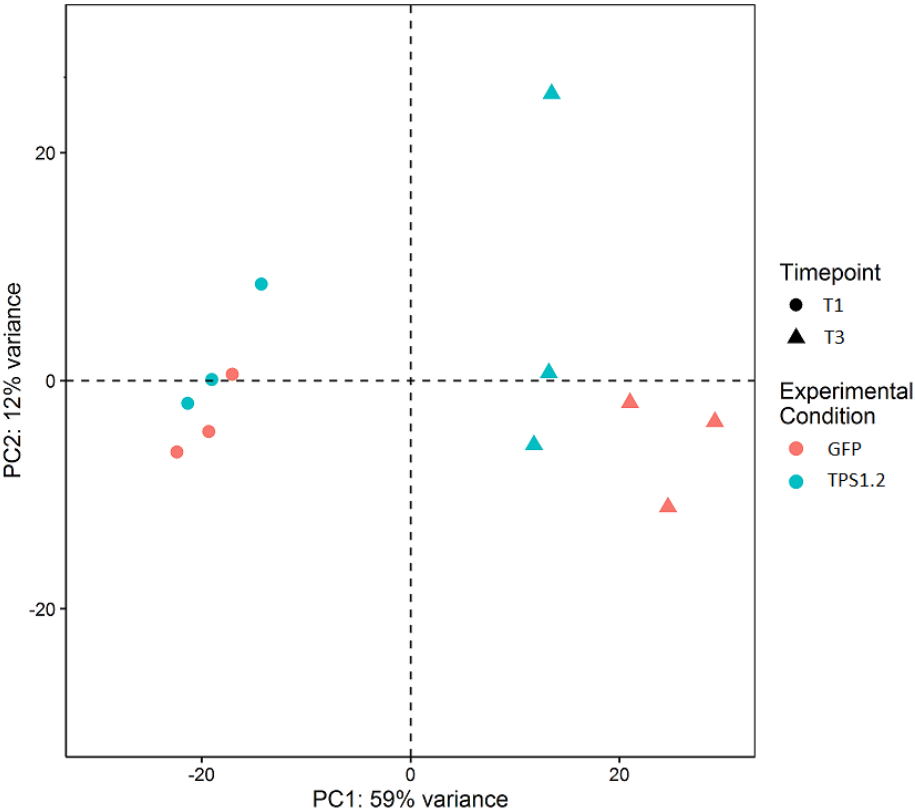

Supplement: S3 Fig — (PDF) [file pone.0168075.s003.pdf]

S4 Fig

A

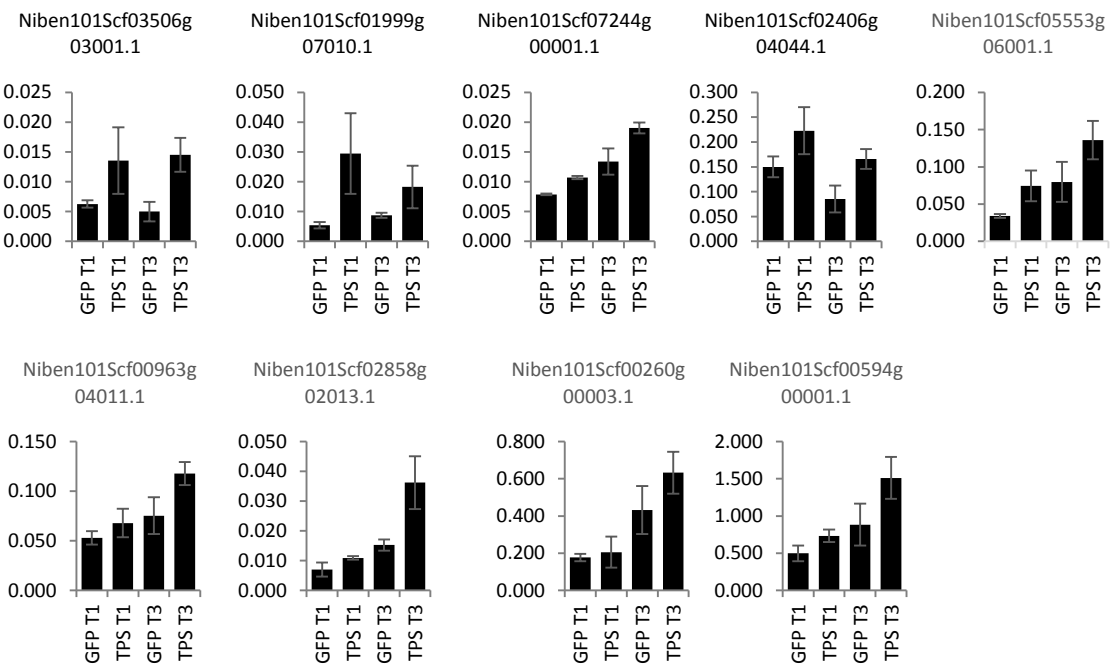

B

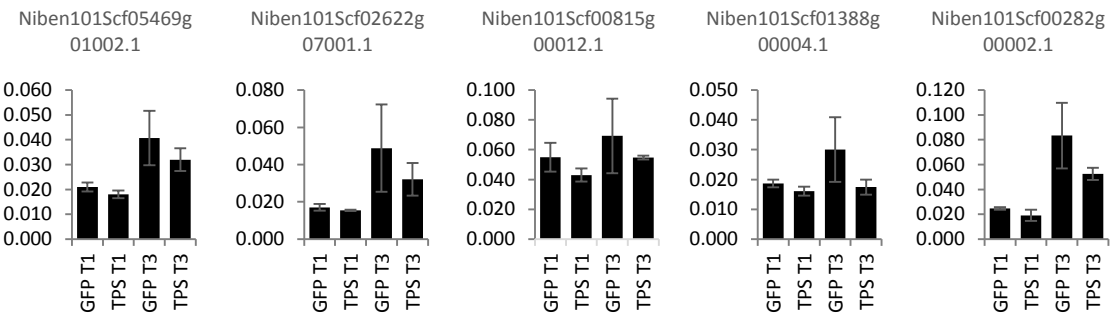

Supplement: S4 Fig — A random selection of differentially expressed transcripts were amplified using sequence-specific oligonucleotide primers. A. Transcripts identified as upregulated in response to elevated TPS1.2a. B. Transcripts identified as repressed in response to elevated TPS1.2a. (PDF) [file pone.0168075.s004.pdf]
