# Supplementary material for: Two Subclasses of Differentially Expressed TPS1 Genes and Biochemically Active TPS1 Proteins May Contribute to Sugar Signalling in Kiwifruit Actinidia chinensis
Source: PLoS One. 2016 Dec 19;11(12):e0168075. doi: 10.1371/journal.pone.0168075 (PMC5167275; doi:10.1371/journal.pone.0168075)
Supplement: S2 Fig — Residues important for Glc6P and UDP-Glc binding are indicated by green and yellow boxes, respectively, and residues important for stabilization of the interaction are indicated by asterisks. (PDF) [file pone.0168075.s002.pdf]

S2 Fig

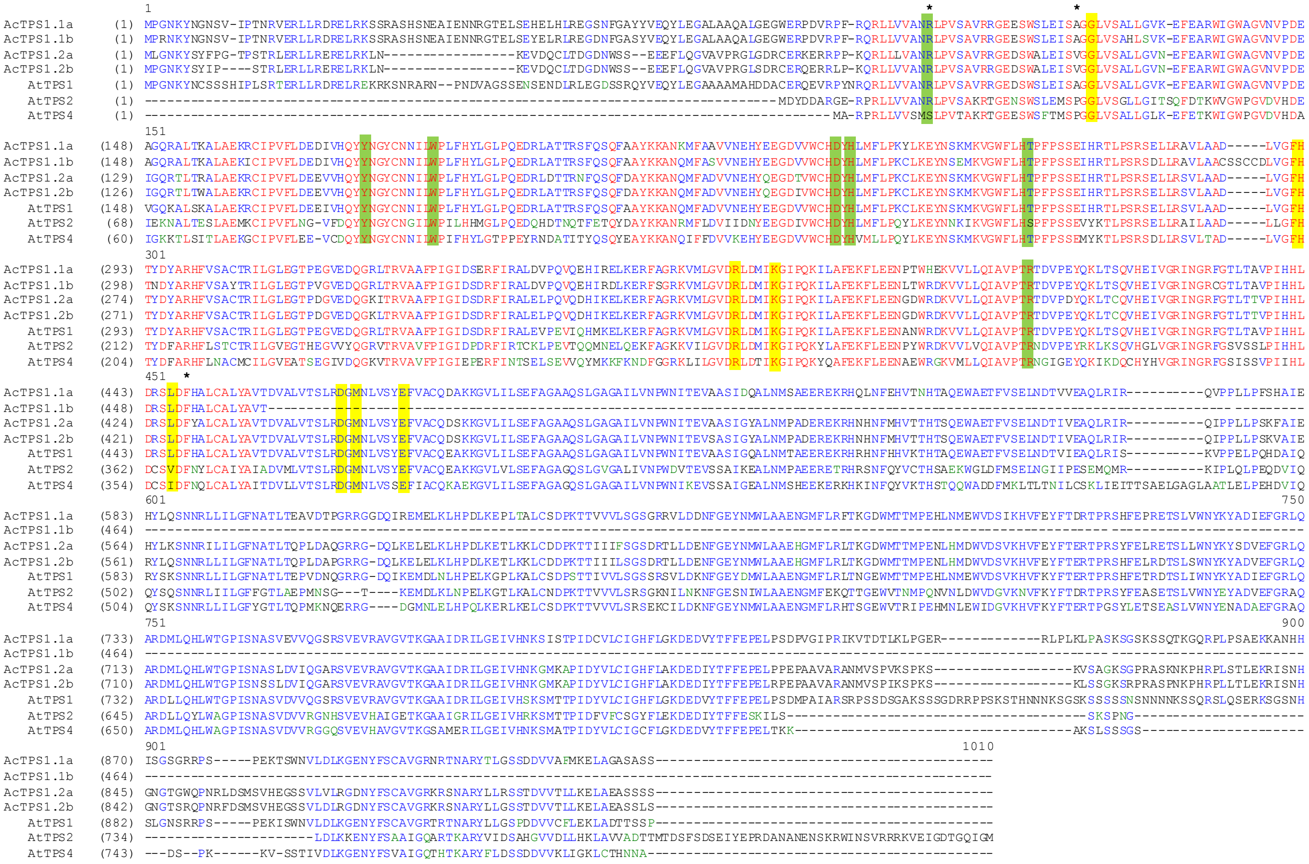
AcTPS1.1a
AcTPS1.1b
AcTPS1.2a
AcTPS1.2b
AtTPS1
AtTPS2
AtTPS4
